# Predictivity of CRP, Albumin, and CRP to Albumin Ratio on the Development of Intensive Care Requirement, Mortality, and Disease Severity in COVID-19

**DOI:** 10.7759/cureus.33600

**Published:** 2023-01-10

**Authors:** Yusuf Uzum, Ebru Turkkan

**Affiliations:** 1 Internal Medicine, Katip Celebi University, Ataturk Training and Research Hospital, Izmir, TUR

**Keywords:** crp-albumin ratio, coronavirus pandemic, covid-19, albumin, crp

## Abstract

Background: The C-reactive protein (CRP) to albumin ratio (CAR) is a new index calculated by dividing CRP by the albumin level. It has been claimed to have predictive value in determining morbidity and mortality in many critical diseases

Aim: In this research, we aimed to elucidate the importance of CRP, albumin, and CAR as parameters that can predict the clinical course in COVID-19 patients.

Materials & method: In this retrospective analysis, the clinical, laboratory, and radiological findings of patients over the age of 18 who were diagnosed with SARS-CoV-2 infection with a positive reverse transcription-polymerase chain reaction (RT-PCR) test were evaluated. Age, gender, laboratory examinations at admission, and CRP and albumin values at the time of diagnosis have been recorded. The relationship of these parameters with the requirement for intensive care, exitus, and serious illness in the clinical follow-up of the patients was investigated. The baseline hospitalization parameters of the patients were compared between the severe and non-severe groups.

Results: Individuals with severe disease had a higher rate of additional disease than those with non-severe disease. It was observed that the mean laboratory values ​​of patients with severe disease had a statistically higher level of D-dimer, CRP, aspartate aminotransferase (AST), platelet distribution width (PDW), CRP-albumin ratio, and ferritin, compared to mild to moderate cases (p<0.05). The rate of additional disease in deceased patients was higher than in patients who were alive (p<0.05). The CAR value was found to be moderately predictive in our study revealing the severity of the disease, and the possibility that the severity of the disease might be higher in patients with a CAR value above 21.47.

Conclusion: The results of this study revealed that CAR is a potential parameter in distinguishing critically ill COVID-19 patients in need of intensive care. Therefore, one can say that CAR is an important biomarker in clinically determining COVID-19.

## Introduction

Severe acute respiratory syndrome of coronavirus was first seen in December 2019 in Wuhan, China. The coronavirus epidemic 2019 (COVID-19) has been caused by SARS-CoV-2. Studies from all over the globe on the diagnosis and clinical findings of the disease have been reported in a very short time and clinicians directed their clinical practice in light of this information. Laboratory examinations at the time of diagnosis were another valuable piece of information in predicting the clinical course of the disease [1].

The major symptoms of COVID-19 can be elaborated as fever, dry cough, weakness, fatigue, and dyspnea. Amplification of viral RNA by reverse transcription-polymerase chain reaction (RT-PCR) is the gold standard diagnostic test for confirming infection [2]. Prediction of the severity of the disease by the basal biochemical values ​​measured at the hospitalization of the patients has been investigated extensively in each pandemic center. The total blood count on the admission of patients was examined. The C-reactive protein (CRP), also known to be a positive acute phase reactant, and albumin known as a negative acute phase reactant, have been analyzed [3].

The CRP to albumin ratio (CAR) is a new index calculated by dividing CRP by the albumin level. It has been claimed to have predictive value in determining morbidity and mortality in many critical diseases [4-6]. Studies conducted on CAR in COVID-19 patients show that it can be a prognostic indicator of disease severity [7,8].

Intensive care requirement is a common problem in COVID-19 patients, especially with acute respiratory distress syndrome and multiple organ failure [9]. Therefore, detecting the patient in need of intensive care in the early term and distinguishing critical and non-critical patients is crucial for the prognosis of the disease. The role of the systemic inflammatory response has become increasingly important in the pathophysiology of COVID-19 infection and many studies have been conducted to identify the predictive value of various inflammatory parameters such as interleukin-6, D-dimer, neutrophil/lymphocyte ratio, fibrinogen, and procalcitonin [9,10].

The CRP increase is a positive acute phase reactant, and it responds to infection, trauma, tissue damage, cardiovascular disease, and other inflammatory events [11]. In COVID-19 patients, the increase in CRP is associated with the severity of the disease [12]. On the other hand, albumin is a negative acute phase reactant that reflects the nutritional status of the patient as well as surgery, burns, and inflammation via a decrease in plasma levels [13]. Decreased serum albumin levels are common in COVID-19 patients and hypoalbuminemia has been shown to correlate with mortality [14].

The CRP to albumin ratio has the potential to represent both the inflammatory response and the nutritional state of the patient simultaneously. Therefore, it is a more reliable dynamic parameter rather than CRP or serum albumin alone. Park et al. reported that CAR has been proven to be more accurate than CRP alone in predicting the mortality of critically ill patients in a 28-day period [15]. The CRP to albumin ratio has recently been used as a prognostic biomarker in various inflammatory conditions [16]. It is considered a useful index to predict mortality in critical illnesses such as sepsis and septic shock. [17].

Dai et al. published a study of 419 COVID-19 RT-PCR-positive patients and stated that a significant difference was found between individuals with severe and stable disease in terms of CRP (p<0.001) ​​and albumin (p<0.001) values [18]. Additionally, Karakoyun et al. elaborated on the statistically significant relationship between CRP (p<0.001), CAR (p<0.001), and aspartate aminotransferase (AST) (p=0.047) in the serious disease group compared with mild to moderate individuals in a cohort of 197 subjects [8].

This research aimed to elucidate the importance of laboratory parameters that can predict the clinical course in patients who are clinically and radiologically thought to have COVID-19 infection with a positive RT-PCR result. Among these parameters, CRP, albumin, and CAR have been evaluated concerning the predictivity of these parameters in terms of the development of intensive care requirements, mortality, and severity of the disease.

## Materials and methods

In this retrospective analysis, we aimed to evaluate the clinical, laboratory, and radiological findings of patients over the age of 18 who were diagnosed with SARS-CoV-2 infection with a positive RT-PCR test. Patients were hospitalized in the internal diseases COVID-19 ward of İzmir Katip Celebi University Ataturk Training and Research Hospital. The ethics committee approval was granted for this study (approval no. 0040). The study complied with the Declaration of Helsinki and informed consent was obtained from all participants.

Age, gender, laboratory examinations at admission, and CRP and albumin values at the time of diagnosis of all patients have been recorded. The CRP to albumin ratio was calculated. The examinations done in the first 24 hours of admission were analyzed. The relationship of these parameters with the requirement for intensive care, exitus, and serious illness in the clinical follow-up of the patients was investigated. The baseline hospitalization parameters of the patients were compared between the severe and non-severe groups.

Patients with COVID-19 were considered to have severe illness if they had a saturation of peripheral oxygen (SpO2) <90% on room air at sea level, arterial oxygen pressure (PaO2)/fraction of inspired oxygen (FiO2) <300 mm Hg, and respiratory rate >30 breaths/minute. The non-severe group included symptomatic patients without signs of hypoxia or pneumonia, as well as patients with moderate symptoms of pneumonia such as dyspnea, cough, and fever, with SpO2 ≥ 90%. The severity of the disease was evaluated based on the COVID-19 guideline published by the WHO (World Health Organization), and mild to moderate illnesses were evaluated as non-severe.

The complete blood count (CBC) measurements were based on impedance-based cell counting operating on the Coulter principle in UniCel DxH 800 hematology analyzer (Beckman Coulter, Miami, FL, USA). The other biochemical parameters were determined using a turbidimetric method. The COVID-19 diagnosis was confirmed via RT-PCR (Bio-Speedy SARS-CoV-2 RT-qPCR kit, Bioeksen R&D Technologies Ltd, Istanbul, Turkey) of viral nucleic acids from the throat and nasal swab samples.

Inclusion & exclusion criteria

Patients ≥18 years old who were hospitalized in our institution with positive RT-PCR results were included in the study.

Patients with missing medical data; individuals who had another infection other than SARS-CoV-2 that may affect the CRP and albumin values and thus the CAR; a liver disease that might cause low albumin value, proteinuria; renal disease such as nephrotic syndrome that required albumin replacement were not included in the study.

Statistical analysis

Patient data collected within the scope of the study were analyzed with the SPSS version 23.0 (IBM Corp., Armonk, NY, USA). Frequency and percentage for categorical data, and mean and standard deviation for continuous data were given as descriptive values. The receiver operating characteristic (ROC) analysis was performed to determine the determinant of CAR value on disease severity, intensive care requirement, and mortality. Independent sample t-test was utilized for comparisons between groups, and chi-square or Fisher's exact test was used for comparison of categorical variables. The results were considered statistically significant when the p-value was less than 0.05.

## Results

Within the scope of the study, a total of 272 patients, 144 (52.9%) male and 128 (47.1%) female subjects were included in this retrospective analysis. The distribution of demographic and clinical findings of the participants according to the severity of the disease is given in Table 1.

**Table 1:** Comparison results of baseline characteristics between severity of diseases of COVID-19 patients CRP: C-reactive protein, CAR: C-reactive protein to albumin ratio, AST: Aspartate aminotransferase, ALT: Alanine transaminase, WBC: White blood cell, PLT: Platelet, MPV: Mean platelet volume, Hb: Hemoglobin, RDW: Red cell distribution width

Variables	Total (N=272)	Non-Severe (n=158)	Severe (n=114)	p-value
	n (%) or Mean±SD	n (%) or Mean±SD	n (%) or Mean±SD	
Age, Year	65±14	63±15	69±13	<0.001
Gender				0.685
Male	144 (52.9)	82 (51.9)	62 (54.4)	
Female	128 (47.1)	76 (48.1)	52 (45.6)	
Comorbidity	197 (72.4)	96 (60.8)	101 (88.6)	<0.001
Diabetes Mellitus	110 (40.4)	50 (31.6)	60 (52.6)	<0.001
Hypertension	165 (60.7)	77 (48.7)	88 (77.2)	<0.001
Chronic Renal Failure	43 (15.8)	19 (12)	24 (21.1)	0.065
Coronary Artery Disease	70 (25.7)	34 (21.5)	36 (31.6)	0.061
Congestive Heart Failure	48 (17.6)	23 (14.6)	25 (21.9)	0.158
Laboratory				
D-Dimer	929.3±1690.6	629.3±951.7	1345.1±2301.2	<0.001
Albumin	3.4±0.5	3.6±0.5	3.2±0.4	<0.001
CRP	87.3±64	66.4±57.7	116.4±61.2	<0.001
CAR	27±21.3	19.9±18.7	36.9±20.7	<0.001
AST	49±52.8	43.1±45.8	57.3±60.5	0.028
ALT	34.6±42.5	34.1±50.8	35.3±27.4	0.814
WBC	8199.5±4814	8052.6±5000.1	8403.2±4557.4	0.554
Neutrophile	6189.8±4395.8	5998.9±4498	6454.4±4255.5	0.400
Lymphocyte	1221±1276.9	1353±1572	1038±647.2	0.044
PLT	257.1±108.5	254.5±111.3	260.7±104.7	0.645
MPV	11.3±1.4	11.2±1.4	11.4±1.3	0.174
PDW	12.9±2.4	12.6±2.3	13.2±2.4	0.029
Hb	12.7±6.4	13±8.3	12.3±1.8	0.341
RDW	14.3±2.5	14.1±2.4	14.5±2.5	0.170
Ferritin	502.8±528.4	413.3±458.8	626.8±592.1	0.002
Procalcitonin	1.4±5.6	1.5±6.5	1.2±4.1	0.632
Intensive Care Unit (ICU)	63 (23.2)	11 (7)	52 (45.6)	<0.001
Last Status				<0.001
Alive	221 (81.3)	148 (93.7)	73 (64)	
Death	51 (18.8)	10 (6.3)	41 (36)	

When the table was examined, 58.1% of the cases (n=158) did not have a severe disease while 41.9% (n=114) had a severe COVID-19. There was a statistically significant difference between the age distributions of the patients according to the severity of the disease (p<0.05). The age of the severe disease group was higher than the non-severe group.

There was a statistically significant difference between the disease severity groups in terms of comorbidity (p<0.05). Those with severe disease had a higher rate of additional disease than those with non-severe disease. There was a statistically significant difference between the two groups in laboratory parameters (p<0.05). It was observed that the mean laboratory values ​​of those with severe disease had higher levels of D-dimer, CRP, AST, PDW, CAR, and ferritin compared to mild to moderate cases.

The distribution of demographic and clinical findings according to the survival status of the participants is given in Table 2.

**Table 2:** Comparison results of baseline characteristics between last status of COVID-19 patients CRP: C-reactive protein, CAR: C-reactive protein to albumin ratio, AST: Aspartate aminotransferase, ALT: Alanine transaminase, WBC: White blood cell, PLT: Platelet, MPV: Mean platelet volume, Hb: Hemoglobin, RDW: Red cell distribution width

Variables	Total (N=272)	Alive (n=221)	Death (n=51)	p-value
	n (%) or Mean±SD	n (%) or Mean±SD	n (%) or Mean±SD	
Age, Year	65±14	64±14	72±13	<0.001
Gender				0.758
Male	144 (52.9)	118 (53.4)	26 (51)	
Female	128 (47.1)	103 (46.6)	25 (49)	
Comorbidity	197 (72.4)	152 (68.8)	45 (88.2)	0.009
Diabetes Mellitus	110 (40.4)	88 (39.8)	22 (43.1)	0.782
Hypertension	165 (60.7)	128 (57.9)	37 (72.5)	0.077
Chronic Renal Failure	43 (15.8)	28 (12.7)	15 (29.4)	0.006
Coronary Artery Disease	70 (25.7)	51 (23.1)	19 (37.3)	0.056
Congestive Heart Failure	48 (17.6)	31 (14)	17 (33.3)	0.002
Severity of disease				<0.001
Non-Severe	158 (58.1)	148 (67)	10 (19.6)	
Severe	114 (41.9)	73 (33)	41 (80.4)	
Laboratory				
D-Dimer	929.3±1690.6	747.6±1121.2	1716.5±3030	<0.001
Albumin	3.4±0.5	3.5±0.5	3.1±0.5	<0.001
CRP	87.3±64	80.3±63.4	117.8±58	<0.001
CAR	27±21.3	24.1±20.1	39.8±21.7	<0.001
AST	49±52.8	42.2±40.4	78.5±82.8	0.004
ALT	34.6±42.5	34.1±44.7	36.7±31.7	0.701
WBC	8199.5±4814	8105.3±4890	8607.8±4492.7	0.503
Neutrophile	6189.8±4395.8	6119.4±4413.5	6494.9±4348.1	0.583
Lymphocyte	1221±1276.9	1253.2±1372.9	1081.3±720.5	0.387
PLT	257.1±108.5	257.6±104.6	255±125	0.876
MPV	11.3±1.4	11.2±1.3	11.6±1.5	0.037
PDW	12.9±2.4	12.9±2.4	12.7±2	0.563
Hb	12.7±6.4	12.6±2.1	13.4±14.3	0.682
RDW	14.3±2.5	13.8±1.9	16.3±3.4	<0.001
Ferritin	502.8±528.4	493.7±545.7	542.2±448.7	0.556
Procalcitonin	1.4±5.6	0.6±1.7	4.9±11.9	0.006
Intensive Care Unit (ICU)	63 (23.2)	17 (7.7)	46 (90.2)	<0.001

When the table was examined, 81.3% (n=221) of the cases were alive, while 18.7% (n=51) were deceased. The rate of additional diseases in deceased patients was higher than in patients who were alive (p<0.05). In addition, the rate of patients with severe disease was higher than those who survived (p<0.05). There was a statistically significant difference between the two groups in some of the laboratory parameters (p<0.05). It was observed that the mean laboratory values ​​of the deceased individuals were higher than the individuals who were alive.

The ROC curve results for examining the differential effect of individuals in terms of CAR according to disease severity are given in Table 3.

**Table 3:** ROC analysis results of CAR for the severity of disease in COVID-19 patients CAR: C-reactive protein to albumin ratio, ROC: Receiver operating characteristic, AUC: Area under the curve, CI: Confidence interval

Risk Factor	AUC (95% CI)	Cut-off	p-value	Sensitivity (%)	Specificity (%)
CAR	0.756 (0.701-0.806)	>21.47	<0.001	76.3	66.5

**Figure 1:**
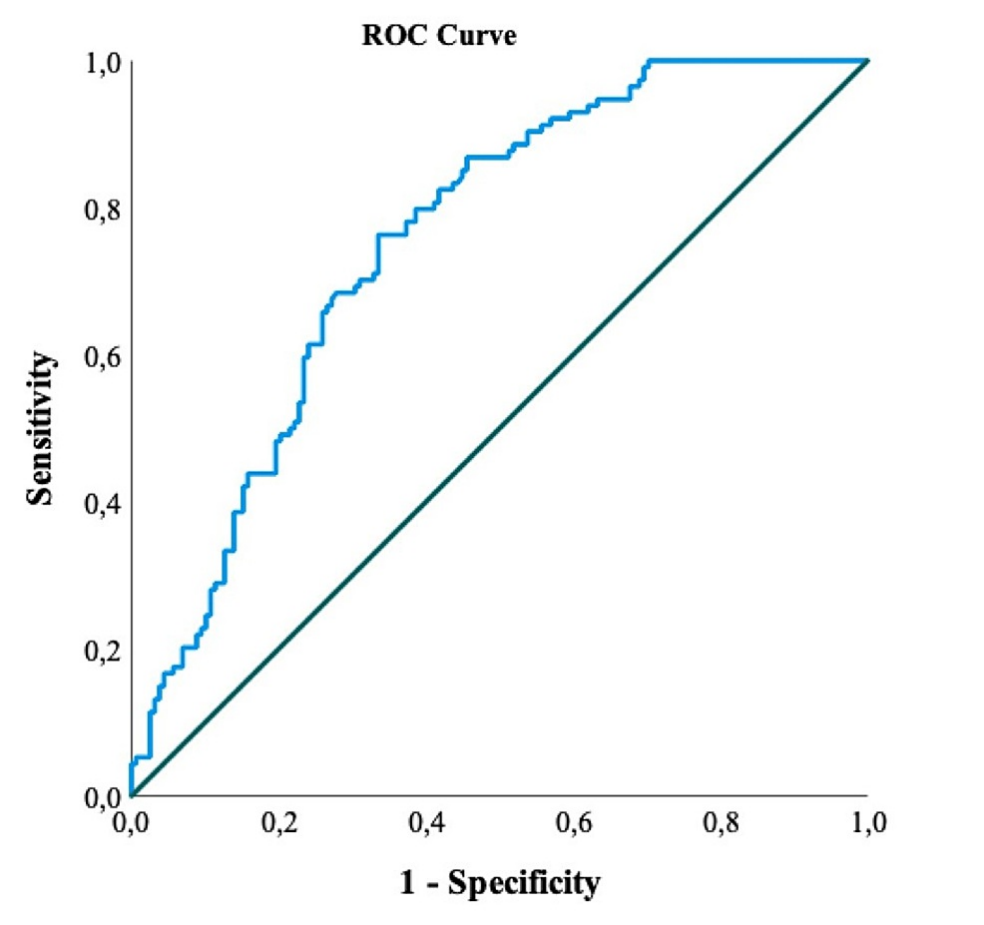
ROC curve of CAR for the severity of disease in COVID-19 patients CAR: C-reactive protein to albumin ratio, ROC: Receiver operating characteristic

While the area under the curve (AUC) for CAR was 75.6%, the cut-off value was determined as 21.47. The AUC shows the statistical significance of the discrimination ability of the diagnostic test. Since the diagnostic test evaluated in our study was disease severity, the value found for CAR was determined to have a moderate (70% to 80%) discrimination ability (Figure 1).

Table 4 denotes the ROC curve results to examine the differential effect of individuals in terms of CAR according to their final status.

**Table 4:** ROC analysis results of CAR for the mortality of COVID-19 patients CAR: C-reactive protein to albumin ratio, ROC: Receiver operating characteristic, AUC: Area under the curve, CI: Confidence interval

Risk Factor	AUC (95% CI)	Cut-off	p-value	Sensitivity (%)	Specificity (%)
CAR	0.721 (0.664-0.774)	>21.52	<0.001	80.4	55.7

**Figure 2:**
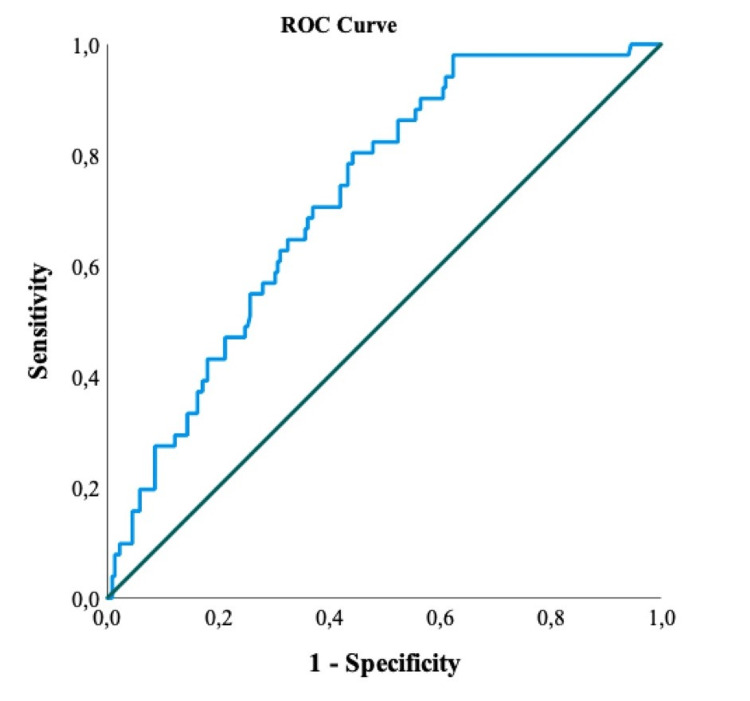
ROC curve of CAR for the mortality of COVID-19 patients CAR: C-reactive protein to albumin ratio, ROC: Receiver operating characteristic

While the AUC for CAR was 72.1%, the cut-off value was determined to be 21.52. The AUC determines the statistical significance of the discrimination ability of the diagnostic test. Since the diagnostic test evaluated in our study was mortality, the value found for CAR was determined to have a moderate (70% to 80%) discrimination ability (Figure 2).

The mortality rate in those with severe disease levels was higher than in those with non-serious disease levels. This difference was statistically significant (p<0.05).

The ROC curve results examining the differential effect of CAR in terms of the requirement for intensive care are shown in Table 5.

**Table 5:** ROC analysis results of CAR for the intensive care requirement of COVID-19 patients CAR: C-reactive protein to albumin ratio, ROC: Receiver operating characteristic, AUC: Area under the curve, CI: Confidence interval

Risk Factor	AUC (95% CI)	Cut-off	p-value	Sensitivity (%)	Specificity (%)
CAR	0.744 (0.679-0.810)	>25.71	<0.001	71.4	65.1

**Figure 3:**
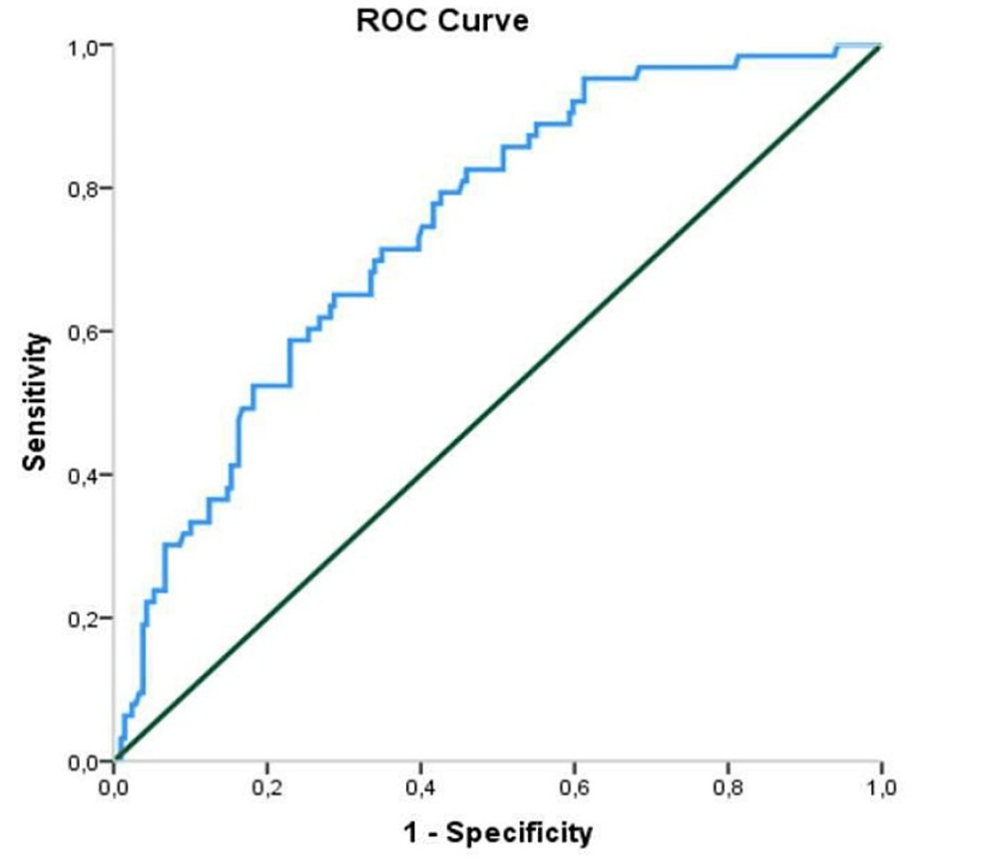
ROC curve of CAR for the intensive care requirement of COVID-19 patients CAR: C-reactive protein to albumin ratio, ROC: Receiver operating characteristic,

While the AUC for CAR was 74.4%, the cut-off value was determined to be 25.71. The AUC indicates the statistical significance of the discrimination ability of the diagnostic test. Since mortality was the last condition evaluated in our study, the value found for CAR was found to be moderate (70% to 80%) of discrimination ability (Figure 3).

## Discussion

The COVID-19 infection has a wide clinical spectrum from asymptomatic infection to critical illness. While clinical conditions requiring follow-up in the intensive care unit such as acute respiratory distress syndrome and multi-organ failure develop in patients with critical COVID-19, the prognosis of non-critical patients is much better [1,9]. For this reason, it is important to detect the patient in need of intensive care as early as possible and to distinguish critical and non-critical patients for timely treatment before the disease progresses. The role of the systemic inflammatory response is becoming increasingly important in the pathophysiology of COVID-19 infection. In this context, many studies have focused on the predictive value of various inflammatory parameters such as interleukin-6, D-dimer, neutrophil/lymphocyte ratio (NLR), fibrinogen, and procalcitonin in determining the severity of COVID-19 disease [10].

Viruses infect lymphocytes and increase their destruction, causing lymphopenia and a decrease in serum albumin, which is a negative acute-phase reactant, while inducing an increase in CRP and ferritin levels, which are positive acute-phase reactants [12,13]. In addition, lactate dehydrogenase (LDH) enzyme levels, which are found in many tissues in the body, may also increase in cases where cell damage due to viral infection develops. In most of the studies conducted for SARS-CoV-2 infection, lymphopenia, low serum albumin levels, and increased LDH, AST, ALT, creatine phosphokinase-MB (CK-MB), and troponin levels are observed [17]. In a study carried out in the Netherlands, it was stated that a scale called “corona score”, which includes age, gender, CRP, ferritin, LDH, lymphocyte count, neutrophil count, and chest X-ray results, can be created and used to differentiate SARS-CoV-2 positive and negative patients [19]. The median of this score was 4 in SARS-CoV-2 negative patients and 11 in positive ones, and 96% sensitivity and 95% specificity were observed for these cut-off values. The negative predictive value of the model was calculated as 88% and the positive predictive value as 96% [19]. In our study, 41.9% of the individuals were classified as severe patients and those with severe disease had a higher rate of additional disease. In terms of laboratory parameters, patients with severe disease had higher levels of D-dimer, CRP, AST, PDW, CRP-Albumin ratio, and ferritin compared to mild to moderate cases.

C-reactive protein is a positive acute phase reactant that increases in response to infection, trauma, tissue damage, cardiovascular disease, and other inflammatory events [11]. It has previously been shown that CRP increased in COVID-19 patients and this increase is associated with the severity of the disease [20]. Albumin, which is a negative acute phase reactant, reflects the nutritional status of the patient and decreases in conditions such as surgery, burns, and inflammation [13]. Decreased serum albumin levels are common in COVID-19 patients, and previous studies have shown that hypoalbuminemia is correlated with increased mortality [14,20].

The CAR has the potential to represent both the inflammatory response and the nutritional status of the host simultaneously. Therefore, its use is a more reliable dynamic index than CRP alone or serum albumin alone. In a study by Park et al., CAR has been shown to be more accurate than CRP alone in predicting 28-day mortality in critically ill patients [15]. The CAR has also been recently defined as a prognostic biomarker in various inflammatory conditions [16]. It has been accepted as a useful index in predicting mortality in critical diseases such as sepsis and septic shock [17]. However, studies on its role in SARS-CoV-2 infection are small and few in number [8, 21]. As expected, CAR in severe COVID-19 patients was significantly higher when compared to healthy individuals. To prevent unnecessary or inappropriate use of health resources, we determine the need for intensive care in patients with COVID-19, and we believe that CAR can be a predictive index. The CRP to albumin ratio value was found to be moderately predictive in our study revealing the severity of the disease, and the possibility that the severity of the disease might be higher in patients with a CAR value above 21.47.

Neutrophil lymphocyte ratio (NLR), platelet lymphocyte ratio (PLR), and CAR have high sensitivity and specificity in demonstrating inflammation. Studies have shown that NLR, PLR, and CAR are independent prognostic indicators in many diseases [22-24]. In many studies conducted during the COVID-19 pandemic, it has been emphasized that the increase in CAR and NLR can be a biomarker in evaluating the COVID-19 prognosis and distinguishing mild/moderate clinical picture from severe clinical picture [25,26]. In a meta-analysis study, it was stated that NLR and PLR could be used as independent prognostic markers in severe COVID-19 patients [27]. In support of the studies mentioned above, in our study, CAR was found to be significantly higher in severe patients followed in the intensive care unit. These findings suggest that a wider range of inflammatory processes are triggered in severe patients with comorbidities followed up in the intensive care unit, thus the prognosis may be worse in these patients. In our study, 41 subjects (n=41/114) in the severe group, and 10 patients (n=10/158) in the non-severe group were deceased. It can be said that this is the clinical confirmation of the above data. The CAR value was found to be moderately predictive of patients admitted to the intensive care unit, and the possibility that the severity of the disease might be higher in patients with a CAR value above 25.71.

The main limitation of the study could be attributed to its retrospective nature. Additionally, the comorbid diseases of the patients have been inquired about in the intensive care unit, and the stage of the comorbidities has not been documented.

## Conclusions

In conclusion, CAR is a practical, inexpensive, and easily accessible test that can be used routinely. The results of this study revealed that CAR is a potential parameter in distinguishing critically ill COVID-19 patients in need of intensive care. Therefore, one can say that CAR is an important biomarker in clinically determining COVID-19.
